# Effects of Yoga and Mindfulness Programs on Self-Compassion in Medical Professionals during the COVID-19 Pandemic: An Intervention Study

**DOI:** 10.3390/ijerph191912523

**Published:** 2022-09-30

**Authors:** Tomoko Miyoshi, Hiromi Ida, Yoshito Nishimura, Soichiro Ako, Fumio Otsuka

**Affiliations:** 1 Department of General Medicine, Graduate School of Medicine, Dentistry and Pharmaceutical Sciences, Okayama University, Okayama 7008558, Japan; 2 Department of Pharmacy, Okayama University Hospital, Okayama 7008558, Japan; 3 Department of Medicine, John A. Burns School of Medicine, University of Hawai’i, Honolulu, HI 96822, USA

**Keywords:** stress, burnout, yoga, mindfulness, stress in healthcare workers, self-compassion, the COVID-19 pandemic

## Abstract

Stress among healthcare workers (HCWs) increased during the coronavirus disease 2019 pandemic. We aimed to determine whether a yoga and mindfulness program could alleviate burnout and other psychological and physical distress in HCWs, and how this might affect their empathy for patients. A weekly one-hour yoga and mindfulness program was conducted for three months in 2021. Participants were 18 consenting HCWs and, the final analysis included 13 participants. They responded to online questionnaires before and after the program. We measured salivary cortisol levels before and after the program on the first and last days. Self-measured pulse rates (PRs) were taken before and after each session, which decreased significantly in both cases (before, after the first program: 72, 65 bpm, *p* < 0.05; before, after the last program: 75, 66, *p* < 0.05), but salivary cortisol levels did not change. No significant changes were observed in Patient Health Questionnaire-9, Maslach Burnout Inventory, Sense of Coherence, Connor-Davidson Resilience Scale, Self-compassion Scale, or Jefferson Scale of Empathy. However, common humanity, a subscale of self-compassion, increased significantly (before the first program: 5.6, after the last program: 6.5, *p* < 0.05), and over-identification decreased significantly (7.9, 6.7, *p* < 0.01). Yoga and mindfulness programs may help improve the sense of common humanity and reduce over-identification in HCWs.

## 1. Introduction

More than two years have passed since the onset of the coronavirus disease 2019 (COVID-19) pandemic, which exposed healthcare workers (HCWs) worldwide to a significant amount of stress, and possibly burnout [1,2]. Our previous study also showed that nurses and doctors working in emergency rooms, especially those treating patients with COVID-19, were at high risk of burnout [3,4]. Additionally, during the pandemic, physicians faced the double burden of managing the increasing COVID-19 cases and keeping abreast of frequently changing infection-prevention policies [5]; the same is likely true for other HCWs. Compared to pre-pandemic levels, the prevalence of anxiety and depression was higher among HCWs and the general public during the COVID-19 pandemic [6]. Risk factors include being female, nurse, low socioeconomic status, being at high risk of contracting COVID-19, and social isolation [6]. Hospitals with HCWs presenting these risk factors must establish intervention programs to alleviate their psychological distress.

When physicians become physically ill, the performance of the healthcare system declines [7]. Burnout among internal medicine residents is associated with suboptimal patient care practices [8], and the depersonalization dimensions of physician burnout are associated with lower patient satisfaction and longer recovery times after discharge [9]. Burnout has also been negatively associated with empathy in HCWs [10]. Thus, burnout among HCWs affects patient care, making the physical and psychological well-being of HCWs a critical issue. Before the COVID-19 pandemic, duty hour requirements and short inpatient attending rotations effectively combatted physician burnout. Interventions such as stress management, self-care training, communication skills training, and mindfulness-based approaches were reportedly effective in dealing with physician burnout [11]. Nonetheless, evidence from Japan regarding measures to alleviate burnout and other stressors during the pandemic is scarce.

Mindfulness is a concept defined by Kabat-Zinn as paying attention in a particular way: on purpose, in the present-moment, and nonjudgmentally. Various theories have been proposed as to the underlying mechanisms that effect mindfulness practice. Davidson et al. used electroencephalography to suggest that meditation activates the relative left frontal lobe and is associated with decreased anxiety and negative affect and increased positive affect [12]. Fox et al. also reported that meditation is associated with changes in brain anatomical structures, including the frontal lobes [13].

A review of studies incorporating yoga as an alternative or complementary approach to stress management reported psychological and physical improvements in participants [14]. Mindfulness-based stress reduction, such as yoga, reduced the average visits people make to their primary care physician as primary prevention of disease [15,16]. The blood-pressure-lowering effect of yoga has been suggested to be a possible effect on autonomic nervous system function by lowering catecholamine levels [16].

Some yoga and mindfulness articles have been reported during the COVID-19 pandemic. For example, yoga practitioners significantly differed in their perceptions of personal control, illness concerns, and the emotional impact of COVID-19. However, this study found no significant difference in the resilience measure [17]. Oral healthcare professionals improved their physical, mental, and quality of life scores with daily yoga practice during the COVID-19 pandemic [18]. Another study of online-yoga-based meditation for healthcare professionals found a correlation between meditation frequency and higher interoceptive awareness [19].

Regarding the effects of yoga and mindfulness programs in Japan, one study found that psychological and physical stress responses improved when yoga sessions were conducted with first-year college nurses working the night shift [20]. Another study found that mindfulness-based yoga programs improved resilience and mental health in older adults [21]. However, there is a dearth of research on the topic yet.

Accordingly, the present study conducted a yoga and mindfulness program for HCWs during the COVID-19 pandemic to improve their well-being and examined whether there were improvements in their burnout, stress tolerance, and self-compassion levels.

## 2. Materials and Methods

### 2.1. Participants

Participants were healthcare professionals working at Okayama University Hospital (800 beds) who consented to this study. They were informed in writing and orally about the study. They were informed that participation in the study was voluntary, and written consent to participate was obtained from the participants. Records were anonymized and all names were coded to maintain the confidentiality of the participants’ data. Analyses and results are presented in a manner that avoids identification and protects the privacy and integrity of the participants. Furthermore, they had no mental illness or obtained permission from their attending physicians in case of mental illness to participate in the mindfulness program. Engagement in the medical care of patients with coronary infections was not assessed.

### 2.2. Study Design

This yoga and mindfulness-based intervention study was conducted during the COVID-19 pandemic—from January to March and May to July 2021—and consisted of a weekly one-hour yoga and mindfulness session. There were different sets of participants in each period.

Before and after each three-month yoga and mindfulness program, web-based surveys were administered to collect data on their demographics and responses to the following instruments: Patient Health Questionnaire-9 (PHQ-9), Maslach Burnout Inventory-Human Services Survey (MBI-HSS), Sense of Coherence-13 (SOC-13), Connor-Davidson Resilience Scale (CD-RISC), Self-compassion Scale, and Jefferson Scale of Empathy (JSE).

### 2.3. Measurement Scales

The PHQ-9 is a nine-item, four-point (0 = not at all, 3 = almost every day) scale used internationally to screen for evidence of depression in the past two weeks. The maximum score is 27, and the Japanese version of the scale has been validated [22,23]. The MBI-HSS is a 25-item scale measuring burnout. It is rated on a seven-point scale ranging from 0 = not at all to 6 = every day. The subscales of the MBI in the original and Japanese versions have a three-factor structure: emotional exhaustion (EE) and physical exhaustion, depersonalization (DP), and personal accomplishment (PA). The reliability of the Japanese version has been verified [24]. Participants with EE scores ≥ 27 or DP scores ≥ 10 were considered to be experiencing burnout. The MBI has been applied in relation to various psychological indicators and HCW burnout [6,25], and it has attracted wide attention as a reliable indicator of burnout in interpersonal service workers [24]. Other stress-related measures include the SOC, which measures the coherence in how people cope with stressful situations and maintain health [26,27]. The CD-RISC is a twelve-item, five-point scale (1 = almost never, 5 = almost always) that measures participants’ stress coping skills [28]. The validity and reliability of the Japanese version of the CD-RISC have been verified for general adults and university students [29]. Self-compassion is important for an individual’s mental and physical well-being under stress [30,31]. The Self-compassion Scale-Short Form is a twelve-item, five-point scale validated in the Japanese version of the scale [32]. Furthermore, HCWs must be able to empathize with their patients [33]. The JSE is a twenty-item, seven-point (1 = strongly disagree, 7 = strongly agree) scale that measures empathy among medical personnel and has been validated in the Japanese version of the scale [34].

### 2.4. Physical Measures

Saliva (Salivet, Sarstedt Company, Nordrhein-Westfalen, Germany) was collected before and after the first and final programs. Saliva was frozen and measured at Yanaihara Laboratory using the Salivary Cortisol EIA Kit (Shizuoka, Japan). Pulse was self-measured and reported before and after each yoga and mindfulness session.

### 2.5. Intervention Program

The yoga and mindfulness program was offered online to limit the possibility of COVID-19 infection, as people could participate individually rather than in a group setting. The program was held for one hour each week, starting at 7:00 or 8:00 p.m. Yoga sessions were conducted by an outsourced qualified instructor, and meditation was led by a research participant who had been a part of multiple meditation programs for at least one day. The basic session structure was as follows:

Basic meditation: five minutes of sitting and breathing meditation;

Yoga with breath awareness: 40 min;

Mindfulness: 15 min.

In the basic meditation session, we practiced zazen (refers to sitting meditation), with gently closed eyes, and took deep breaths, bringing awareness to our breaths. The 40 min yoga session was a combination of standing, seated, and supine yoga, involving postures, breathing, and meditation. The final mindfulness practice involved breath awareness, performing a body scan, and becoming aware of who we are in the present moment.

During the online information session, we informed the participants how to palpate the radial artery and confirmed that they could self-measure it. The participants practiced the maneuver several times in the session.

### 2.6. Statistics

We analyzed the MBI-HSS scores using JMP version 15.1.0 (SAS Institute Inc., Cary, NC, USA) and the PHQ-9, SOC-13, CD-RISC, self-compassion, and empathy scores using BellCurve for Excel (Social Survey Research Information Co., Ltd., Tokyo, Japan). Significance was defined as *p* < 0.05.

## 3. Results

Eighteen participants attended the program; two withdrew for physical illness, one could not participate in more than half of the sessions and left during the program, and two were missing valid questionnaires. Therefore, 13 participants (2 males and 11 females) were included in the analysis. Hence, a statistical analysis based on the participants’ professions was not possible due to the small sample size.

Table 1 shows the general characteristics of the participants. The median age was 48 years. The sample included three physicians, seven nurses, and three healthcare workers from other categories (a speech therapist, medical office worker, and clinical research manager). 

The pulse rate before and after the first and final programs was significantly low because of their physical conditions (before the first program: 72 bpm, after the first program: 65 bpm, *p* < 0.05; before the last program: 75, after the last program: 66, *p* < 0.05) (Table 2). Moreover, there was no significant difference in the change in pulse rate between the first and final sessions (Table 3). Salivary cortisol levels were also not significantly different before and after the first and final yoga and mindfulness sessions (Table 2). Similarly, there were no significant differences in the changes in salivary cortisol levels (Table 3).

Regarding the MBI, there were three and five medical personnel with symptoms of burnout at the beginning and end of the programs, respectively. There were no significant differences in the EE, DP, and PA scores before and after the program. (Table 4). 

As the median age was 49 years, odds ratios were calculated by dividing the age between the two groups. However, no significant differences were found (Table 5).

Table 6 shows the results of the other questionnaires before and after the program. PHQ-9 scores averaged 6.8 before the program and 5.4 at the end of the program, indicating a mild depressive disorder, but no significant differences were found. The number of those scoring four or below increased from two (before the program) to six (by the end of the program).

The results of SOC-13 showed no significant difference, although the average increased from 50 to 56 after the program. The baseline SOC score is 48.3 ± 10.1 in Japan [26]. However, in this study, the number of individuals with a SOC score less than 48.3 was five (before the program began) but decreased to four (by the end of the program).

The average CD-RISC score was 51.9 before the start of the program and 56.0 at the end of the program, which was not significantly different. The mean of a general group in the United States has been reported as 80, while that of the Japanese is 60. Thus, the participants’ scores in this study are lower than those reported by other studies thus far [28].

The average score on the Self-compassion Scale was 39.1 before the program began and 38.6 at the end, showing no significant difference. However, when analyzed by factor, the score of the subscale common humanity increased significantly, while that of over-identification decreased significantly by the end of the program.

The average score on JSE was 76.8 before the program and 81.7 at the end, with no significant difference.

## 4. Discussion

Mindfulness is a meditation technique that cultivates “awareness of the present moment.” [35]. In the current study, the yoga and mindfulness program did not decrease the number of people experiencing burnout and those scoring four or below on the PHQ-9 scale. However, there was no statistically significant difference before and after the program. Burnout among HCWs during the COVID-19 pandemic has been reported globally, including in Japan [1,2]. The health of HCWs affects the quality of healthcare they provide [7]. Thus, the physical and mental health of HCWs is directly linked to the health of the population.

Mindfulness effectively improves negative emotions and compassion fatigue in HCWs [36]. In our study, the yoga and mindfulness program did not affect overall self-compassion, but the score for common humanity, a subscale of the Self-compassion Scale, increased significantly at the end of the program. According to Neff, self-compassion consists of three main components: self-kindness versus self-judgment, common humanity versus isolation, and mindfulness versus over-identification [37]. In our study, the positive factor of common humanity increased, and the negative factor of over-identification declined. Although we did not find a significant increase in the overall Self-compassion Scale score, but we found an improvement in the scores of the two subscales. Neff et al. have developed a self-compassion-enhancing program helping participants to develop self-compassion [38]. Caring for others leads to caring for oneself. Focusing on the development of self-compassion by healthcare providers using Mindfulnes Based Stress Reduction and other mindfulness interventions is expected to reduce stress and increase the effectiveness of clinical care. [39] A program focused on self-compassion for HCWs in the COVID-19 pandemic may be effective and warrants further research.

The present study implemented yoga and mindfulness programs during the pandemic, but no changes were observed in participants’ scores on PHQ-9, MBI-HSS, SOC-13, CD-RISC, Self-compassion Scale, and JSE. However, the mean values of SOC-13, CD-RISC, and JSE increased after the program, and further validation may better show the program’s effectiveness. Burnout among physicians due to work stress has been reported in recent years. For instance, one study found that 25–60% of physicians felt frustrated with work and that poor physician health could decline the overall quality of healthcare [7]. Thus far, mindfulness programs, including meditation, have been studied for their effects on the mind and body. They are now being introduced in actual patient care [39]. Yoga, including mindfulness programs, has also proved helpful in alleviating burnout in HCWs [17,33,39]. Burnout during the COVID-19 pandemic has been attributed to (i) limited hospital resources, (ii) the threat of exposure to the virus as an occupational hazard, (iii) long shifts, (iv) disrupted sleep patterns, (v) poor work–life balance, (vi) subsequent heightened dilemmas regarding patient duties versus fear of exposure to family members, (vii) neglect of personal and family needs with an increased workload, and (viii) lack of sufficient communication and up-to-date information [40]. Inquiry-based stress reduction (IBSR) has been shown to effectively improve teachers’ well-being, resilience, and burnout [41]. However, its usefulness for healthcare workers during the COVID-19 pandemic should be examined [42].

The yoga and mindfulness program conducted in this study did not improve participants’ psychological indicators, including measures of burnout, in HCWs. There are three possible reasons for this: (i) most previous studies were conducted prior to the pandemic, and overall prevalence of burnout among HCWs might have been lower than what it is currently [4,5,43]; (ii) as this was an online program, participants may have felt that the effectiveness of yoga instructions had decreased after switching from studio to online sessions [44]; and (iii) the frequency of the program may have been insufficient. Prior to the COVID-19 pandemic, ten yoga programs in four months were reported to improve stress and other psychological indicators and cortisol levels [45], online yoga classes were reported to improve the stress coefficient in participants who experienced eleven such classes in four weeks, [46] and yoga-based meditation intervention once a week and meditation on their own outside of the program were reported to improve the burnout index [19].

Regarding changes in physical indices, the effects of the yoga and mindfulness program on the pulse rate before and after the one-hour session were significantly low. Moreover, there was no significant difference in pulse rate and salivary cortisol levels before and after the program, in the first or the last session. Yoga has comparable effects on both physical and mental status, mainly the pulse rate and salivary cortisol, compared to cognitive behavioral therapy [45]. The current study found no changes in the physical information measured during the yoga and mindfulness programs. Previous studies have also reported mixed findings, with some indicating no change in salivary cortisol levels between healthy participants and burnout cases and others finding lower cortisol levels during the day or in the evening [47]. Therefore, the relationship between salivary cortisol levels and stress may require further study.

The COVID-19 pandemic increased stress among HCWs, and weekly yoga and mindfulness interventions did not improve scores on the PHQ-9, MBI-HSS, SOC-13, CD-RISC, Self-compassion Scale, and JSE, as was the case before the COVID-19 pandemic. There are the limitations of self-care addressed by individual HCWs, political measures and hospital management implications need to be reconsidered for the quality of care and continuity of the healthcare system.

The present study has some limitations. The sample size was small and, thus, does not entirely reflect the situation of the general medical professionals. In addition, the participants had an affinity for yoga and mindfulness programs, which may not be true for all medical personnel.

## 5. Conclusions

During the COVID-19 pandemic, a weekly one-hour yoga and mindfulness program increased common humanity, a subscale of the Self-compassion Scale, and decreased over-identification scores. Because having compassion for others involves compassion for self, during the pandemic, yoga and mindfulness programs may improve self-compassion among medical personnel. Given that HCWs suffer from a high risk of burnout, more research regarding evidence-based effective interventions is warranted to alleviate their psychological distress. Political measures and hospital management implications need to be reconsidered for the quality of care and continuity of the healthcare system during the COVID-19 pandemic.

## Figures and Tables

**Table 1:** General characteristics of participants.

Characteristic	Value	SD	95% CI
Gender, no. (%)			
Female	11 (84.6)		
Male	2 (15.4)		
Age			
Mean	49	8.6	6.2–13.9
Job category, no. (%)			
Nurse	7 (53.8)		
Doctor	3 (23.1)		
Other	3 (23.1)		
Total number of participants	13		

Abbreviations: CI, confidence interval; SD, standard deviation.

**Table 2:** Physical effects of the intervention program.

Pulse (bpm)		Median (IQR)	*t*	*F*	*p* Value	Cohen’s d
First program	Before	72 (66–80)	2.90	12	0.0134	0.94
	After	65 (59–68)				
Last program	Before	75 (72–85)	3.11	12	0.0090	1.00
	After	66 (64–72)				
Cortisol (mg/dL)		Median (IQR)	*t*	*F*	*p* Value	Cohen’s d
First program	Before	0.086 (0.062–0.097)	1.35	12	0.20	0.37
	After	0.073 (0.055–0.084)				
Last program	Before	0.077 (0.057–0.098)	0.59	12	0.57	0.23
	After	0.07 (0.062–0.074)				

Abbreviations: IQR, interquartile range. *p* value is calculated with two-sided *t* test.

**Table 3:** Results of physical state before and after the program.

		Median (IQR)	*t*	*F*	*p* Value	Cohen’s d
Δpulse (bpm)	First	6 (2–16)	0.21	12	0.84	0.08
	Last	6 (2–15)				
ΔSalivary cortisol (mg/dL)	First	−0.010 (−0.024 ± 0.012)	1.40	12	0.19	0.50
	Last	0.004 (−0.024 ± 0.007)				

Abbreviations: IQR, interquartile range. *p* value is calculated with two-sided *t* test.

**Table 4:** The Maslach burnout index results before and after the program.

Source		Mean (SD)	*t*	*F*	*p* Value	Cohen’s d
Emotional exhaustion (EE)	Before first program	21 (10.3)	1.58	12	0.14	0.41
	After last program	21.5 (12.8)				
Depersonalization (DP)	Before first program	4.6 (4.0)	0.74	12	0.47	0.23
	After last program	5.5 (4.4)				
Personal accomplishment (PA)	Before first program	23 (8.2)	0.16	12	0.88	0.05
	After last program	25.7 (4.9)				
	Before first program (*n* = 13)	After last program (*n* = 13)				
Measure (No. (%))						
Burnout						
Yes	3 (23.1)	5 (38.5)				
EE > 27	2	5				
DP > 10	2	2				
No	10 (76.9)	8 (61.5)				

Abbreviations: SD, standard deviation. *p* value is calculated with two-sided *t* test.

**Table 5:** Age-dependent prevalence of burnout.

	Variable	OR (95% CI)	*p* Value
	Age		
Last program	≥50 y.o.	1 (Reference)	NA
	<50 y.o.	1.11 (0.11–10.98)	0.923

Abbreviations: CI, confidence interval; NA, not applicable; OR, odds ratio; y.o., years old.

**Table 6:** Results of questionnaire before and after the program.

Source		Mean (SD)	*t*	*F*	*p* Value	Cohen’s d
PHQ-9	Before first program	6.8 (2.4)	1.60	12	0.1360	0.42
	After last program	5.4 (4.3)				
SOC-13	Before first program	50 (6.2)	1.74	12	0.1079	0.69
	After last program	56 (8.8)				
CD-RISC	Before first program	51.9 (14.2)	1.19	12	0.2572	0.36
	After last program	55.9 (7.9)				
Self compassion	Before first program	39.1 (4.8)	0.48	12	0.6433	0.10
	After last program	38.6 (4.3)				
self-kindness	Before first program	6.2 (1.2)	0.90	12	0.3870	0.21
	After last program	6.4 (1.0)				
self-judgement	Before first program	6.5 (1.1)	1.34	12	0.2062	0.52
	After last program	5.9 (1.3)				
common humanity	Before first program	5.6 (1.6)	2.38	12	0.0347	0.53
	After last program	6.5 (1.8)				
isolation	Before first program	6.0 (1.4)	0.26	12	0.7976	0.11
	After last program	5.8 (1.4)				
mindfulness	Before first program	6.8 (1.1)	1.48	12	0.1654	0.50
	After last program	7.3 (0.9)				
over-identification	Before first program	7.9 (1.3)	3.81	12	0.0025	0.91
	After last program	6.7 (1.5)				
Jefferson Empathy	Before first program	76.8 (7.2)	1.50	12	0.1602	0.59
	After last program	81.7 (9.9)				
	Before first program (*n* = 13)	After last program (*n* = 13)				
Measure (No. (%))						
SOC < 53						
Yes	10 (76.9)	5 (38.5)				
No	3 (23.1)	8 (61.5)				
PHQ-9 < 4						
Yes	2 (15.4)	6 (46.2)				
No	11 (84.6)	7 (53.8)				

Abbreviations: SD, standard deviation. *p* value is calculated with two-sided *t* test.

## Data Availability

The data presented in this study are available on request from the corresponding author.
